# Supplementary material for: A global assessment of the drivers of threatened terrestrial species richness
Source: Nat Commun. 2020 Feb 20;11:993. doi: 10.1038/s41467-020-14771-6 (PMC7033199; doi:10.1038/s41467-020-14771-6)
Supplement: Supplementary file 1 — Supplementary Information [file 41467_2020_14771_MOESM1_ESM.pdf]

Supplementary Information for

A global assessment of the drivers of threatened terrestrial species richness

Howard et al.

*Corresponding author. Email: christine.howard@durham.ac.uk

Contents:

Supplementary Methods

Supplementary Figures 1 to 13

Supplementary Tables 1 to 2

Supplementary References

Supplementary Methods

Environmental Covariates

Bioclimatic Variables: Data on four bioclimatic variables (annual mean temperature, temperature seasonality, annual precipitation and precipitation seasonality) were obtained from WorldClim (Version 1.4[1]). Data were available on a 4.5 km grid for the 30-year interval between 1960 and 1990, a period corresponding with the majority of species distribution data collection. We aggregated these data up to a 0.5° Behrman equal area grid, calculating the mean for each of the four bioclimatic variables. These variables have all previously been found to be informative when modelling species richness patterns[2–4].

Elevation Variables: Elevation-related covariates were included in order to capture the demonstrated influence of geophysical heterogeneity on species diversity patterns[5,6]. Elevation data were derived from the Global Multi-resolution Terrain Elevation Data 2010 (GMTED2010[7]). These data were available at a resolution of 7.5 arc-seconds (225 meters). For each 0.5° grid cell we calculated the minimum and standard deviation of elevation (meters).

Land cover diversity: Land cover diversity was selected as a candidate covariate given the role of spatial environmental heterogeneity in increasing available niche space and promoting species coexistence[2]. Land cover data were derived from GlobCover version 2.3 (2009, http://due.esrin.esa.int/page_globcover.php). These data provide global land cover classified into 23 categories at a resolution of 300 m. We estimated the diversity of land cover classes for each 0.5˚grid cell using the Shannon information index[8].

Insularity: When compared with mainland areas, islands are known for their higher levels of endemic, range-restricted species that are potentially more vulnerable to imperillment[9]. To explore the role of islands in promoting concentrations of threatened species richness, we derived a measure of insularity. For this we calculated the total area (km^2) of the land mass of which a grid cell was part. Smaller and more isolated land masses - i.e. islands - have a small value of insularity, whilst grid cells on large continental land mass have a larger value.

Long-term climate stability: The occurrence of endemic, small range and, therefore, extinction prone species has been linked with patterns of climatic stability and change over evolutionary time. Areas with historically stable climates - i.e. areas lacking histories of glaciation - may provide climate refugia and enable the persistence of old and narrowly distributed species. Conversely, climatic instability may promote endemism and speciation through the provision of novel ecological opportunities and habitat fragmentation[10–12]. We therefore explored the potential for climate

stability since the last interglacial period (125,000 years ago) to explain threatened species richness. Palaeo-climate data were made available by the Bristol Research Initiative for the Global Environment (BRIDGE, http://www.bridge.bris.ac.uk/). Comprehensive details of the model used to derive the palaeo-climate data are available elsewhere[13,14]. Data on precipitation and temperature were sampled at 4,000-year intervals. For each temporal transition, the Euclidean distance was calculated between z-transformed temperature and precipitation in bivariate space. The mean Euclidean distance was used as a measure of long-term climate stability in a grid cell, with smaller values indicating more stable climates[15].

Human Influence

Area of anthropogenic land use: Using the land cover data derived from GlobCover version 2.3 (2009, http://due.esrin.esa.int/page_globcover.php) we summed the area of each grid cell that was classified as intensively used by humans. These intensively used land covers included areas of cropland of varying intensity and urbanised areas with artificial surfaces.

Human Influence Index: As the occurrence of intensively used lands can manifest in areas with minimal human settlement[16,17], we also included the Human Influence Index (HII, V2, http://sedac.ciesin.columbia.edu/wildareas/) as an additional measure of human impact. These data are available at a resolution of 1 km^2 and are derived from nine global data layers that cover the period between 1995 and 2004. These data layers cover a range of anthropogenic pressures, both direct and indirect, including human population density, distributions of roads, railways and navigable rivers, and night-time lighting.

Area of protected land: The total area of land receiving some form of protection from transformation was obtained from the World Database on Protected Areas (WDPA: https://www.protectedplanet.net/). We summed the area of land classified as strict nature reserve (Ia), wilderness areas (Ib), national park (II), natural monument or feature (III), habitat or species management area (IV), protected landscape where the interaction of people and nature has produced an area of distinct character with significant ecological and cultural value (V), and areas managed for sustainable use of natural resources (VI). Our expectation was that grid cells with a large area of protected land would support more threatened species than those grids with little to no protection from land transformation, as species could seek refuge in these relatively less disturbed areas[18].

Short-term land cover change: Habitat loss is widely regarded as the primary driver of biodiversity loss[19], with recent changes in the extent of anthropogenic land use linked with changes in species'

extinction risk[20]. To quantify the extent of recent changes in land cover, we obtained data on global land cover from the European Space Agency Climate Change Initiative (ESA CCI, https://www.esa-landcover-cci.org/?q=node/1). These data are available at a spatial resolution of 300 m and consist of annual maps of global land cover between 1992 and 2015. We used these maps to calculate the percentage change in the area of land cover classes associated with anthropogenic land uses between 1992 and 2015 for each of our 0.5° grid cells.

Long-term land cover change: Over the past three centuries, human activities have significantly altered global landscapes, primarily through the conversion of primary habitats to agriculture[21]. If we are to appreciate fully the potential effects of human influences on threatened species richness, we need to consider the magnitude of these past changes. For this we obtained data on geographically explicit changes in croplands between 1700 and 1992. These data were derived from models that use remotely sensed land cover classification data alongside contemporary and historical cropland inventory data to reconstruct historic cropland distributions[21]. These data were available at a 5 min resolution (approximately 10km). We used them to calculate the percentage change in cropland area between 1700 and 1992 for each of our 0.5° grid cells.

Invasive alien species: Invasive alien species are considered one of the greatest threats to biodiversity, second only to habitat loss and fragmentation[22]. For our index of invasive alien species pressures, we obtained data that use information from the Global Invasive Species Database (GSID) and the Centre for Agriculture and Bioscience International's Invasive Species Compendium (CABI ISC) to estimate the number of invasive alien species (IAS) per country[23]. These data include the majority of recorded taxa, including plants, arthropods, amphibians, reptiles, birds and mammals. We intersected these national measures of IAS with the equal area 0.5° grid. For grid cells that intersected more than one country we calculated the area-weighted average IAS across all intersected countries.

Supplementary Figures and Tables

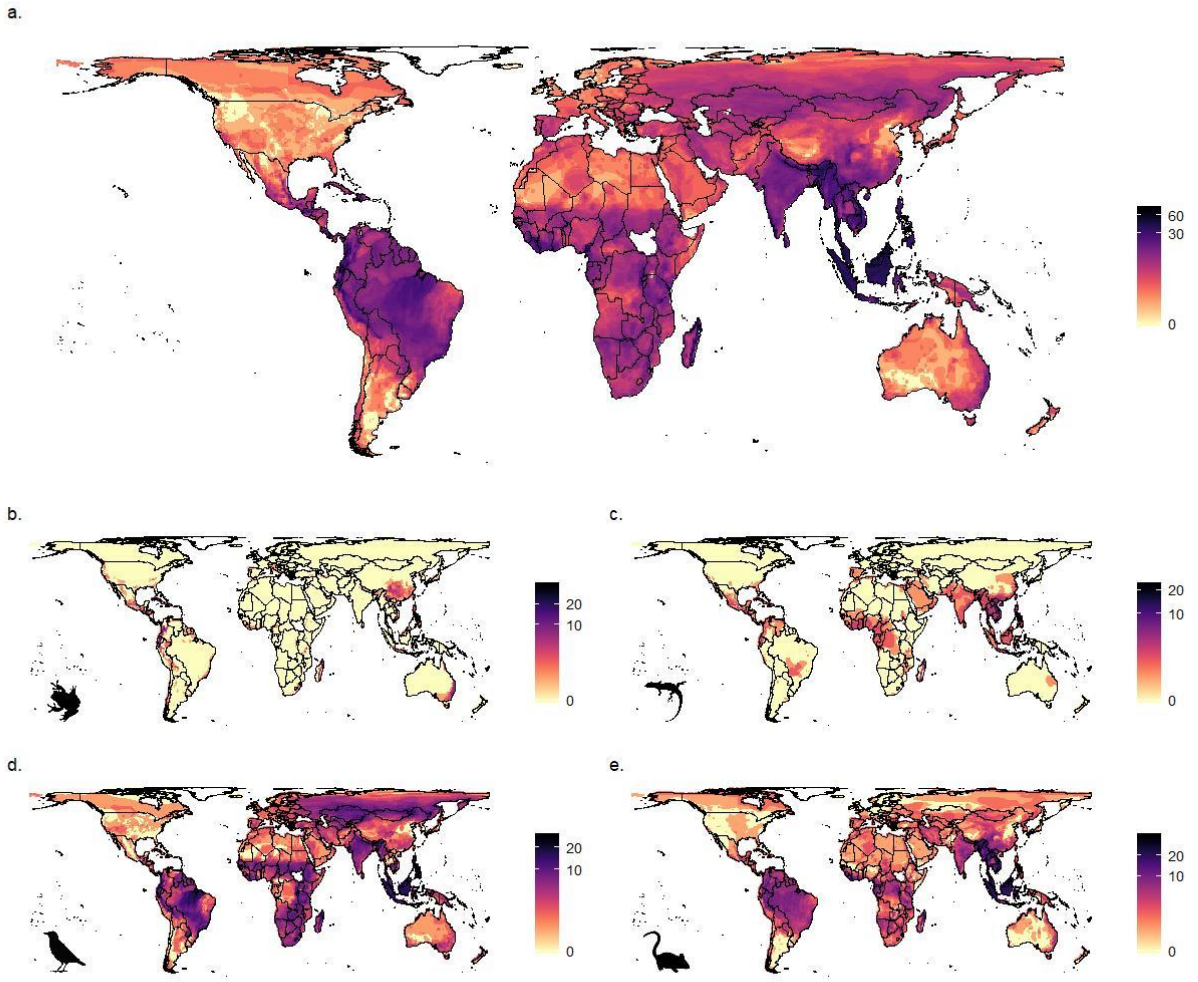

Supplementary Figure 1: Global threatened species richness patterns. Total threatened species richness patterns (i.e. total number of species classified as ‘vulnerable’, ‘endangered’ or ‘critically endangered’ by the IUCN) for a) terrestrial vertebrates, and four separate taxonomic groups: b) amphibians, c) reptiles, d) birds and e) mammals. The colour scale indicates the total number of threatened species in an area. Note different scales used for each panel. Source data are provided as a Source Data file.

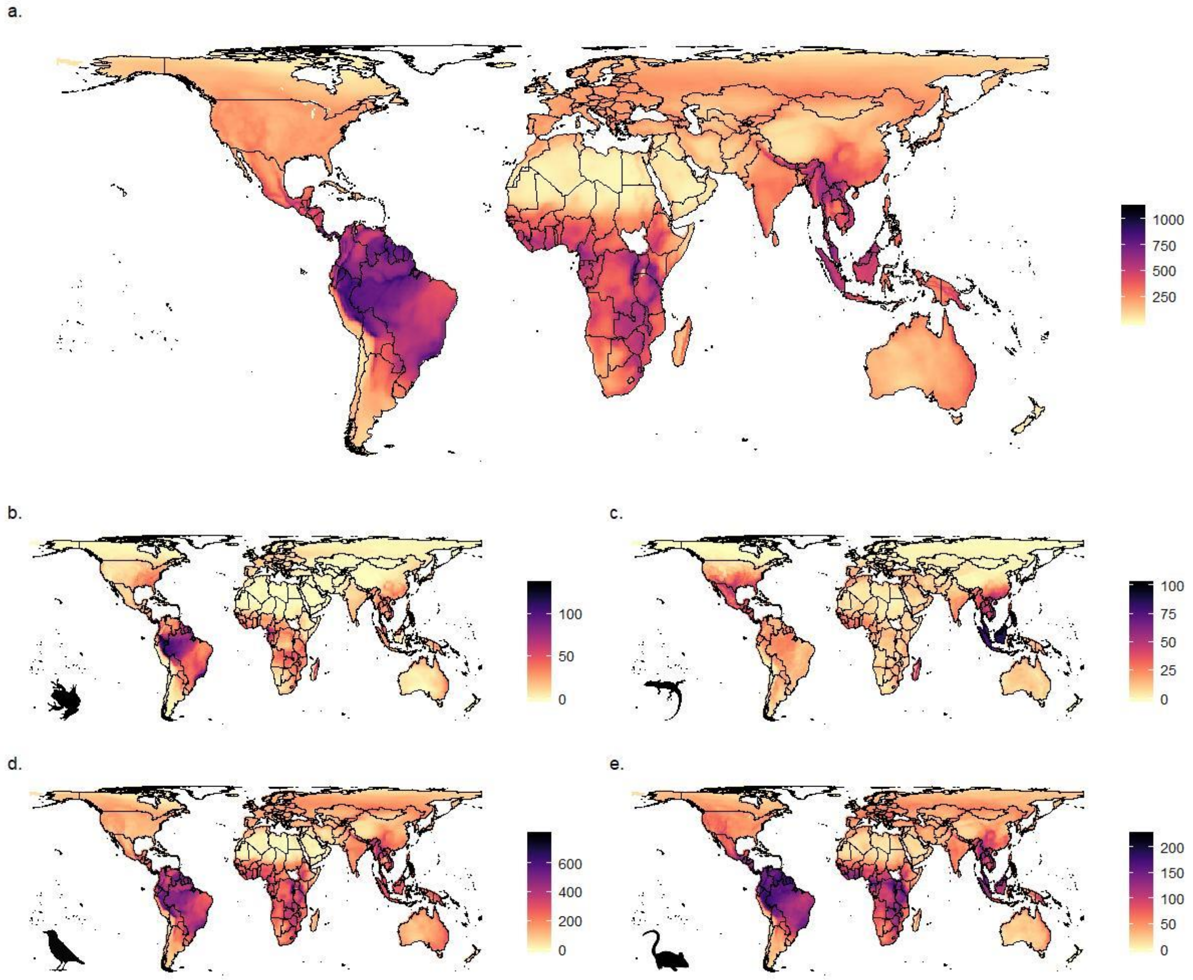

Supplementary Figure 2: Global total species richness patterns. Total species richness patterns for a) terrestrial vertebrates, and four separate taxonomic groups: b) amphibians, c) reptiles, d) birds and e) mammals. The colour scale indicates the total number of species in a grid cell. Note different scales used for each panel. Source data are provided as a Source Data file.

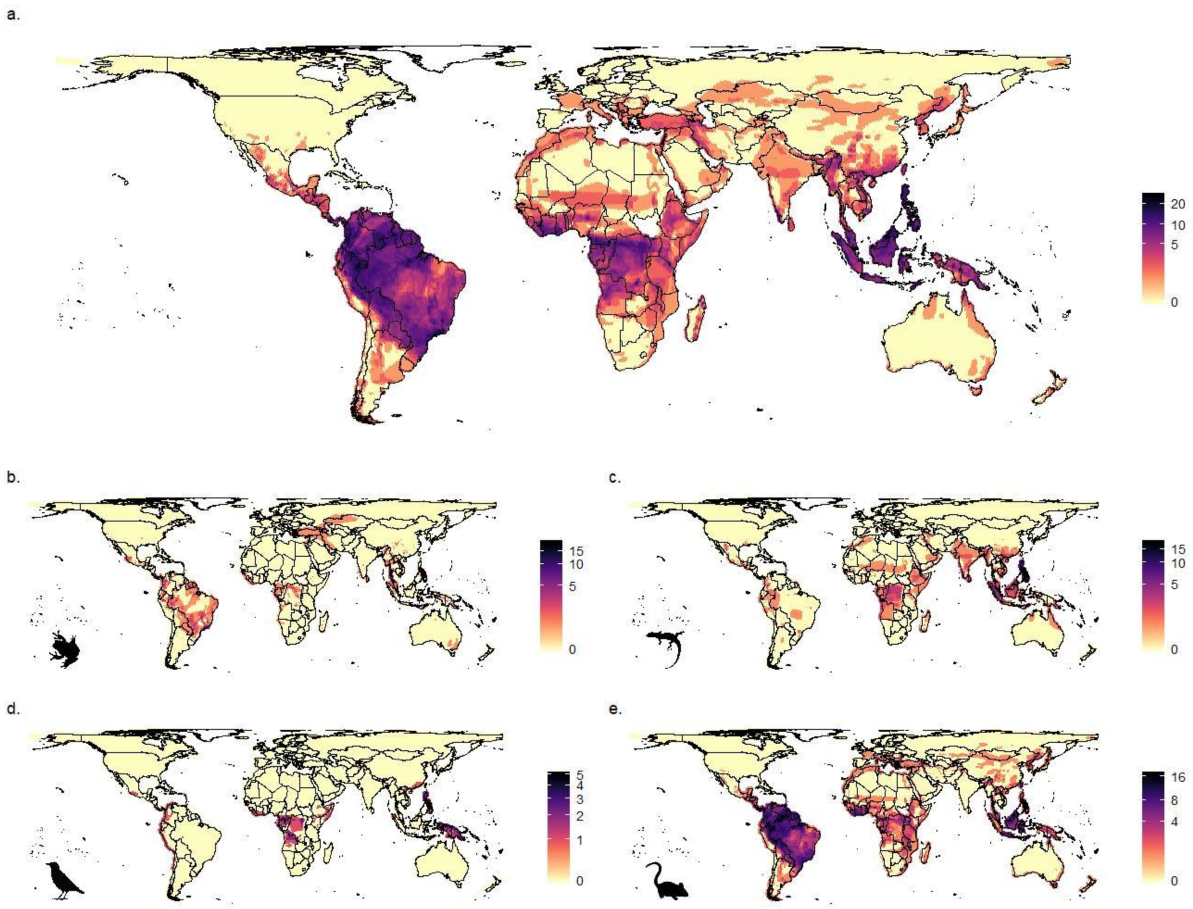

Supplementary Figure 3: Global data deficient (DD) species richness patterns. Data deficient species richness patterns for a) terrestrial vertebrates, and four separate taxonomic groups: b) amphibians, c) reptiles, d) birds and e) mammals. The colour scale indicates the number of data deficient species in a grid cell. Note scales differ between panels. Source data are provided as a Source Data file.

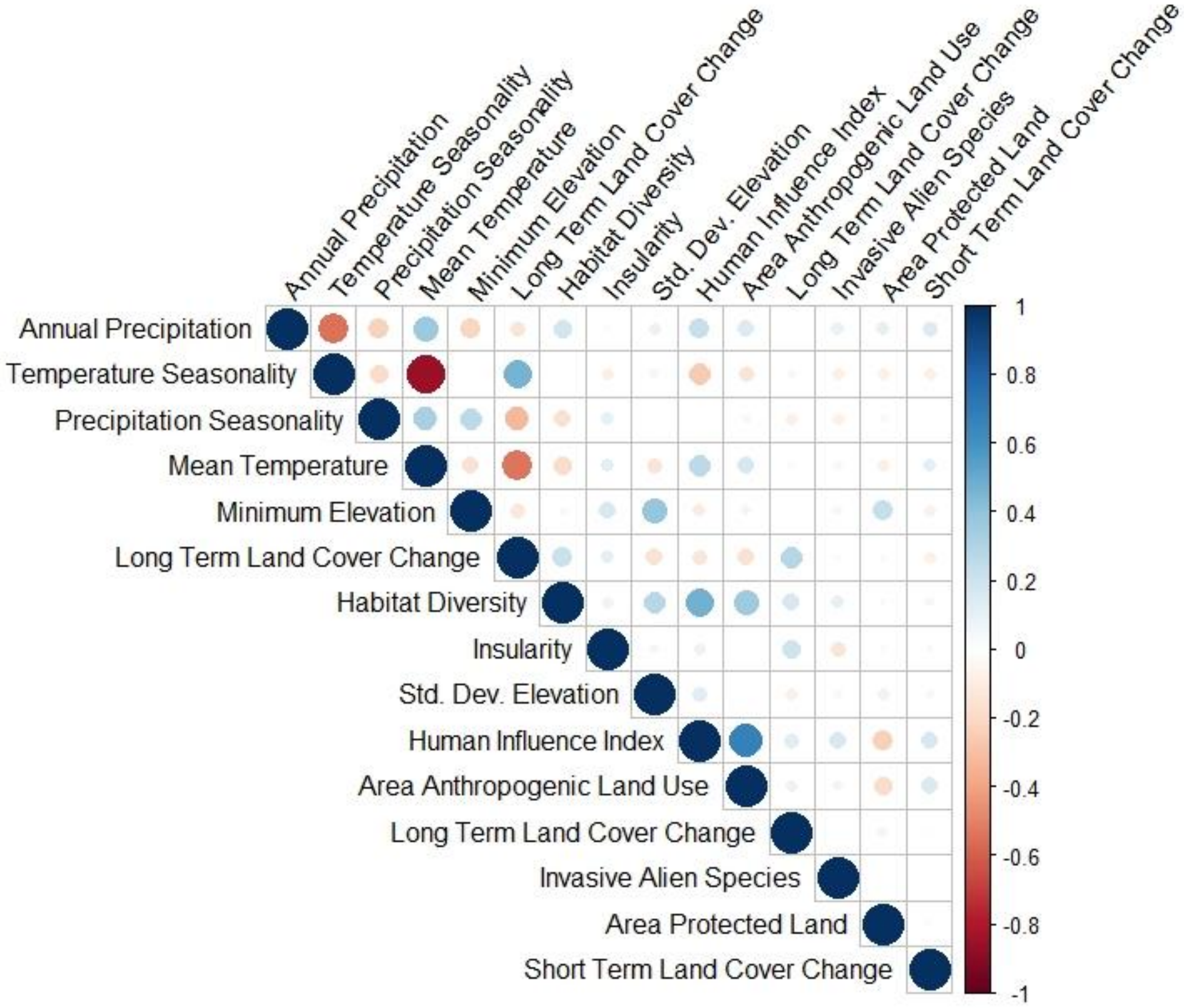

Supplementary Figure 4: Predictor variable correlation plot. Both the colour and size of each point indicate the Pearson's correlation coefficient between each pair of environmental and human impact predictor variables. Source data are provided as a Source Data file.

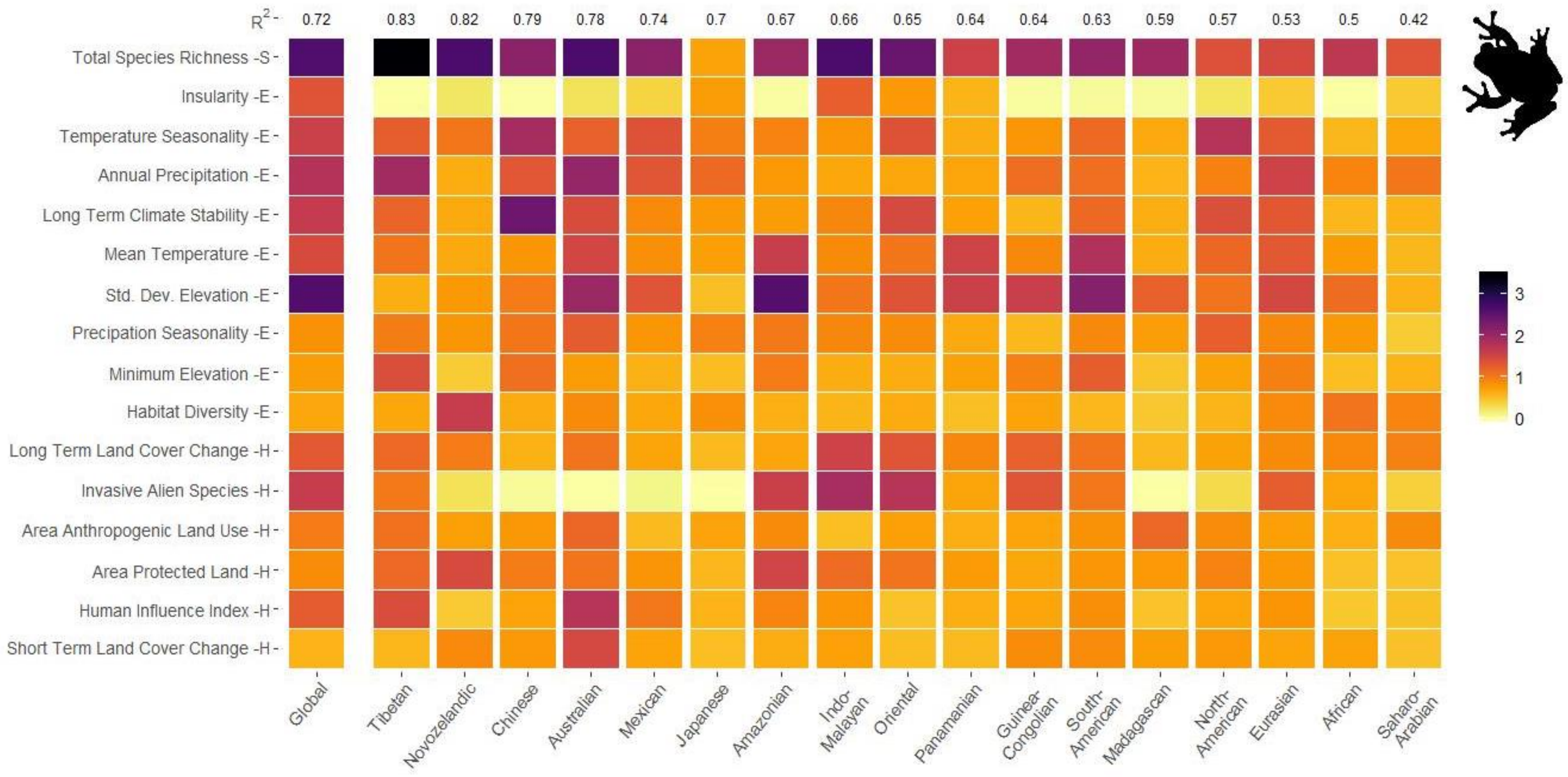

Supplementary Figure 5: Heat map of the importance of variables in predicting threatened amphibian species richness in different zoogeographic regions. Variable classes are indicated by the capital letters on the side of the variable names: Total Species Richness (S), Environmental (E) and Human Impact (H) covariates. The mean importance of each variable from the models of vertebrate threatened species richness is indicated by the yellow (low importance) to black (high importance) gradient. Variables are ordered from top to bottom first by group, and then by their importance in the global model of threatened vertebrate species richness. Average performance of each regional set of models, measured with R^2, is indicated at the top of each column, with regional models ranked by decreasing mean R^2. Source data are provided as a Source Data file.

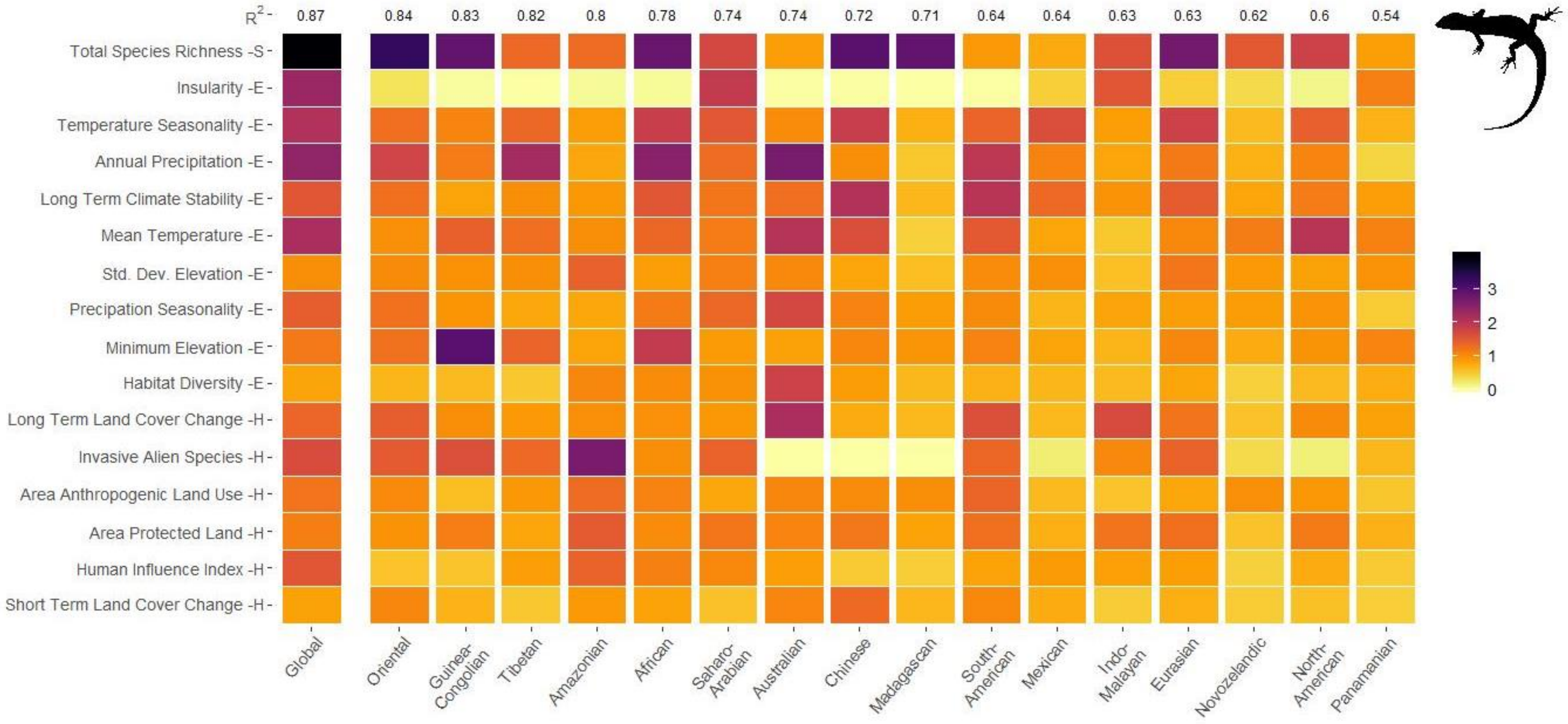

Supplementary Figure 6: Heat map of the importance of variables in predicting threatened reptile species richness in different zoogeographic regions. Variable classes are indicated by the capital letters on the side of the variable names: Total Species Richness (S), Environmental (E) and Human Impact (H) covariates. The mean importance of each variable from the models of vertebrate threatened species richness is indicated by the yellow (low importance) to black (high importance) gradient. Variables are ordered from top to bottom first by group, and then by their importance in the global model of threatened vertebrate species richness. Average performance of each regional set of models, measured with R^2, is indicated at the top of each column, with regional models ranked by decreasing mean R^2. Source data are provided as a Source Data file.

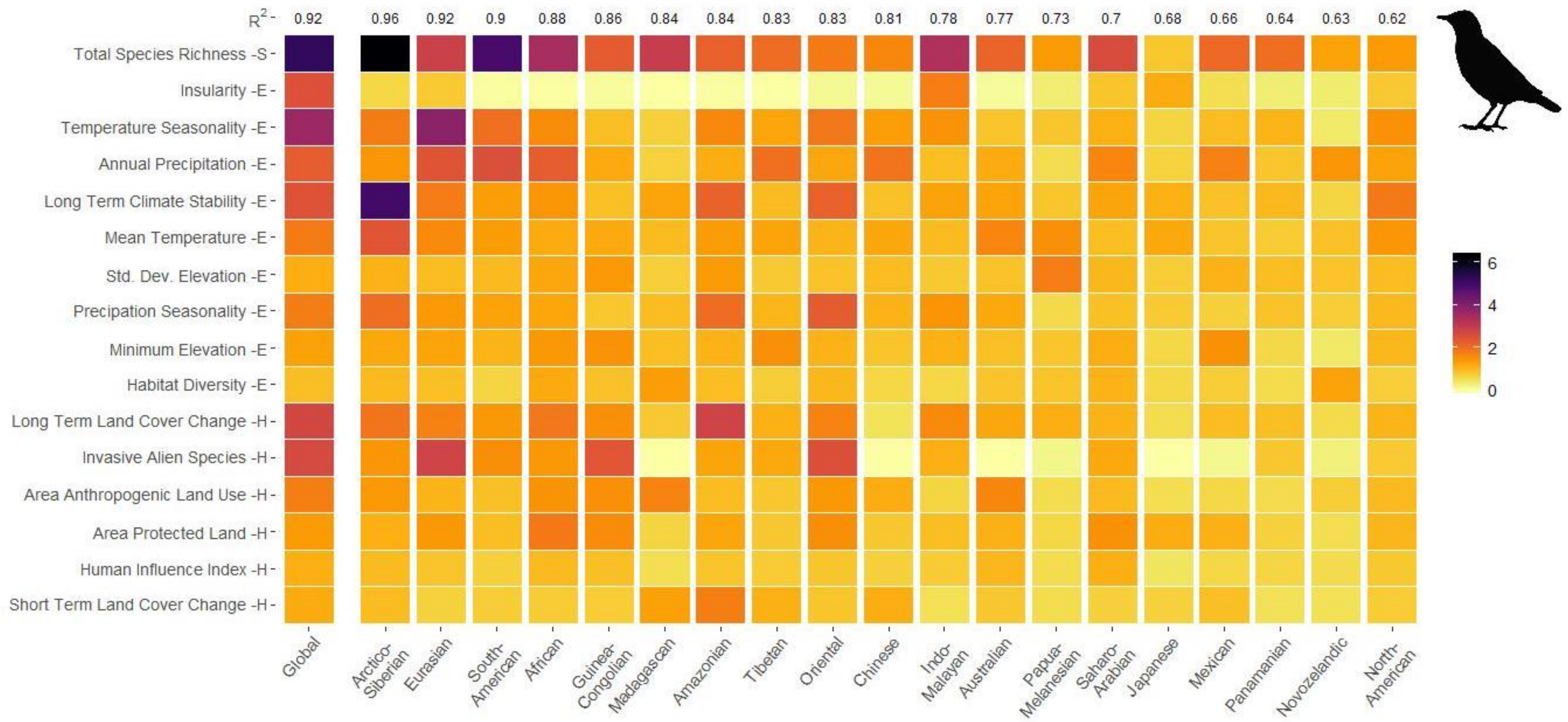

Supplementary Figure 7: Heat map of the importance of variables in predicting threatened bird species richness in different zoogeographic regions. Variable classes are indicated by the capital letters on the side of the variable names: Total Species Richness (S), Environmental (E) and Human Impact (H) covariates. The mean importance of each variable from the models of vertebrate threatened species richness is indicated by the yellow (low importance) to black (high importance) gradient. Variables are ordered from top to bottom first by group, and then by their importance in the global model of threatened vertebrate species richness. Average performance of each regional set of models, measured with R^2, is indicated at the top of each column, with regional models ranked by decreasing mean R^2. Source data are provided as a Source Data file.

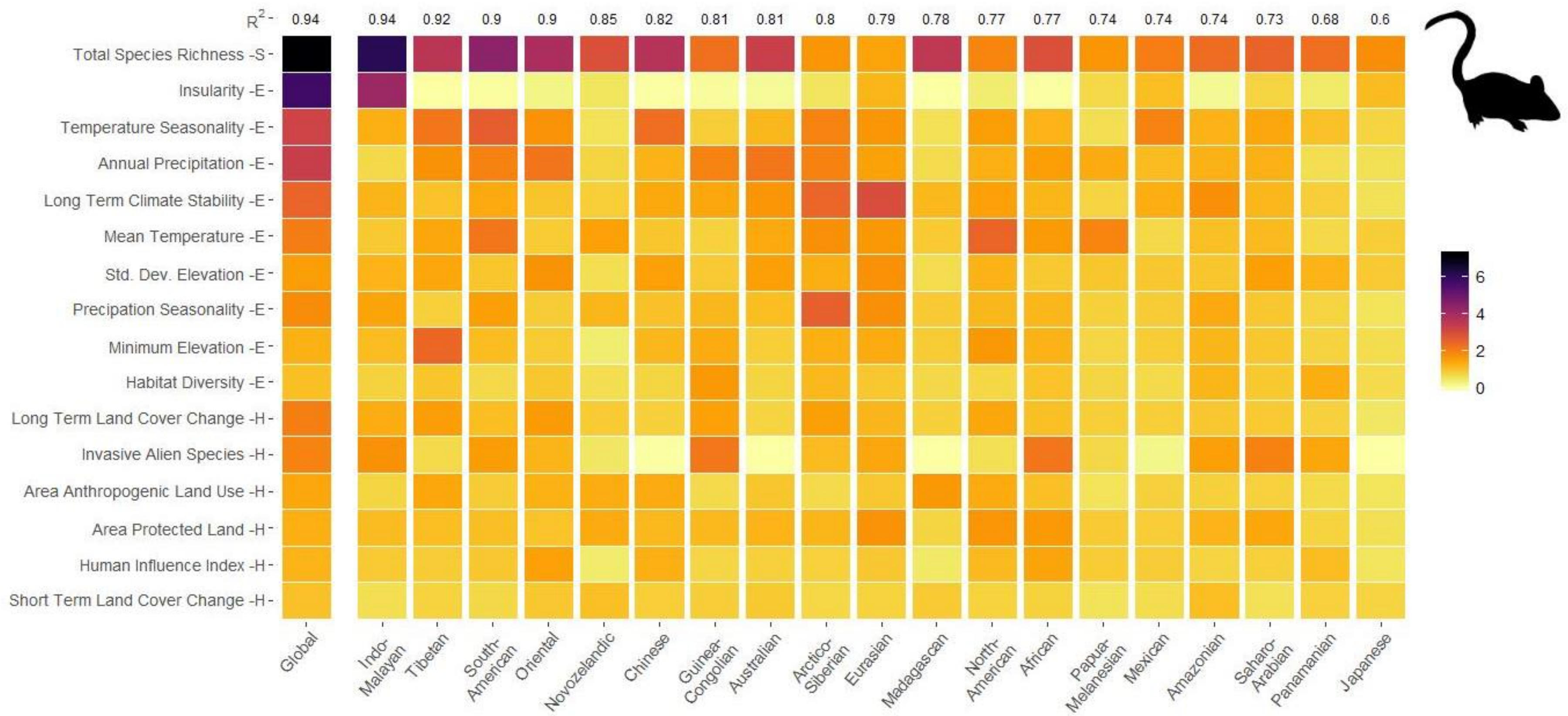

Supplementary Figure 8: Heat map of the importance of variables in predicting threatened mammal species richness in different zoogeographic regions. Variable classes are indicated by the capital letters on the side of the variable names: Total Species Richness (S), Environmental (E) and Human Impact (H) covariates. The mean importance of each variable from the models of vertebrate threatened species richness is indicated by the yellow (low importance) to black (high importance) gradient. Variables are ordered from top to bottom first by group, and then by their importance in the global model of threatened vertebrate species richness. Average performance of each regional set of models, measured with R^2, is indicated at the top of each column, with regional models ranked by decreasing mean R^2. Source data are provided as a Source Data file.

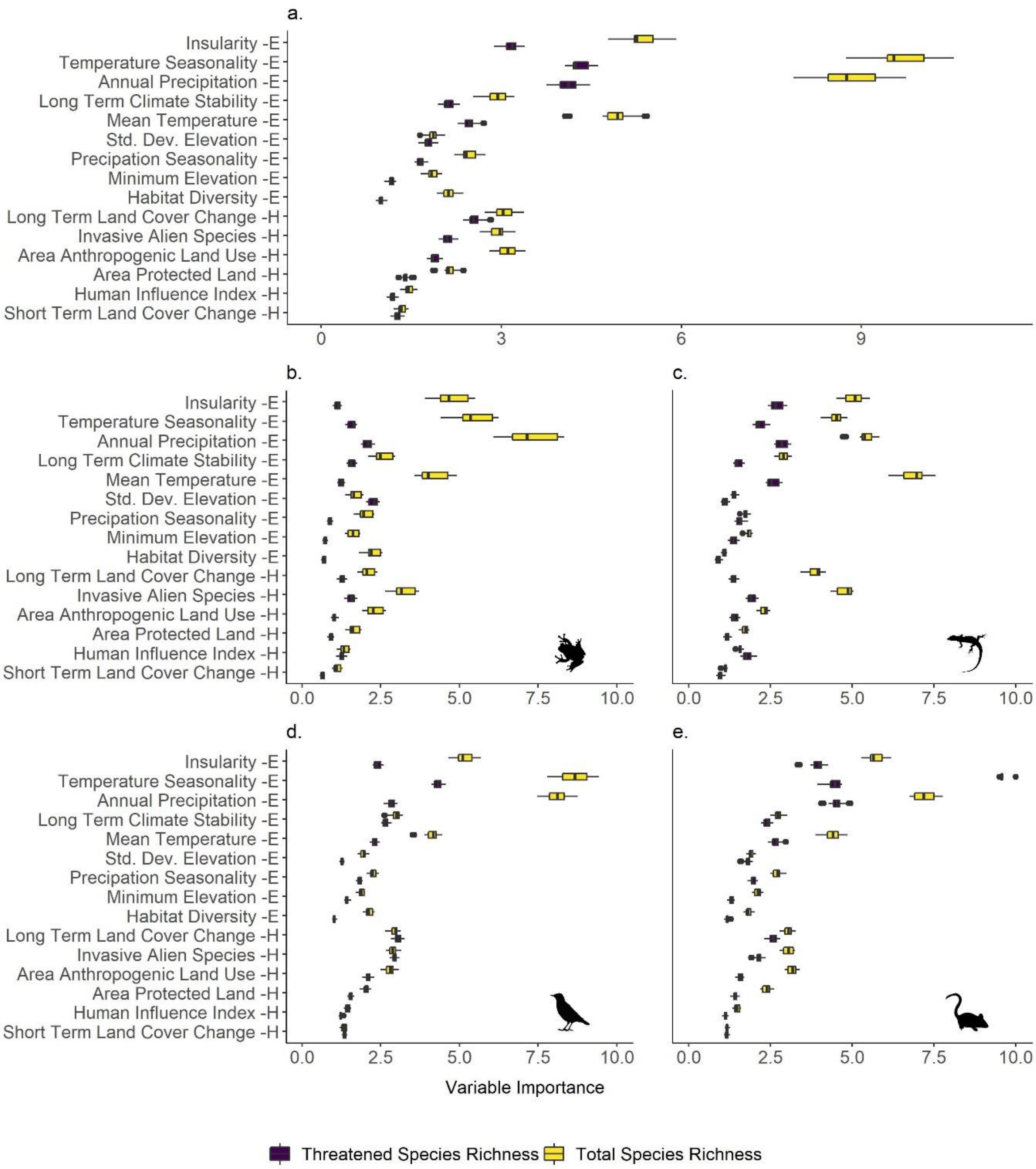

Supplementary Figure 9: Importance of individual variables for predicting global threatened and total species richness. Top panel (a.) indicates the importance of individual variables from the global models of vertebrate species richness, whilst the bottom panels indicate the measures of individual variable importance from the global models of amphibian (b.), reptile (c.), bird (d.) and mammal (e.) species richness. Variables are grouped into broader classes, which are indicated by the capital letters on the side of the variable names: Environmental (E) and Human Impact (H) covariates. Note these models do not included total species richness as a covariate. Bars are shaded according to modelled response: threatened species richness (purple) and total species richness (yellow). Variables are ordered from top to bottom first by group, and then by their importance in the global model of threatened vertebrate species richness. The line across each box indicates the median and the box boundaries indicate the interquartile range (IQR). Whiskers identify extreme data points that are not more than 1.5 times the IQR on both sides; the dots are more extreme outliers. Source data are provided as a Source Data file.

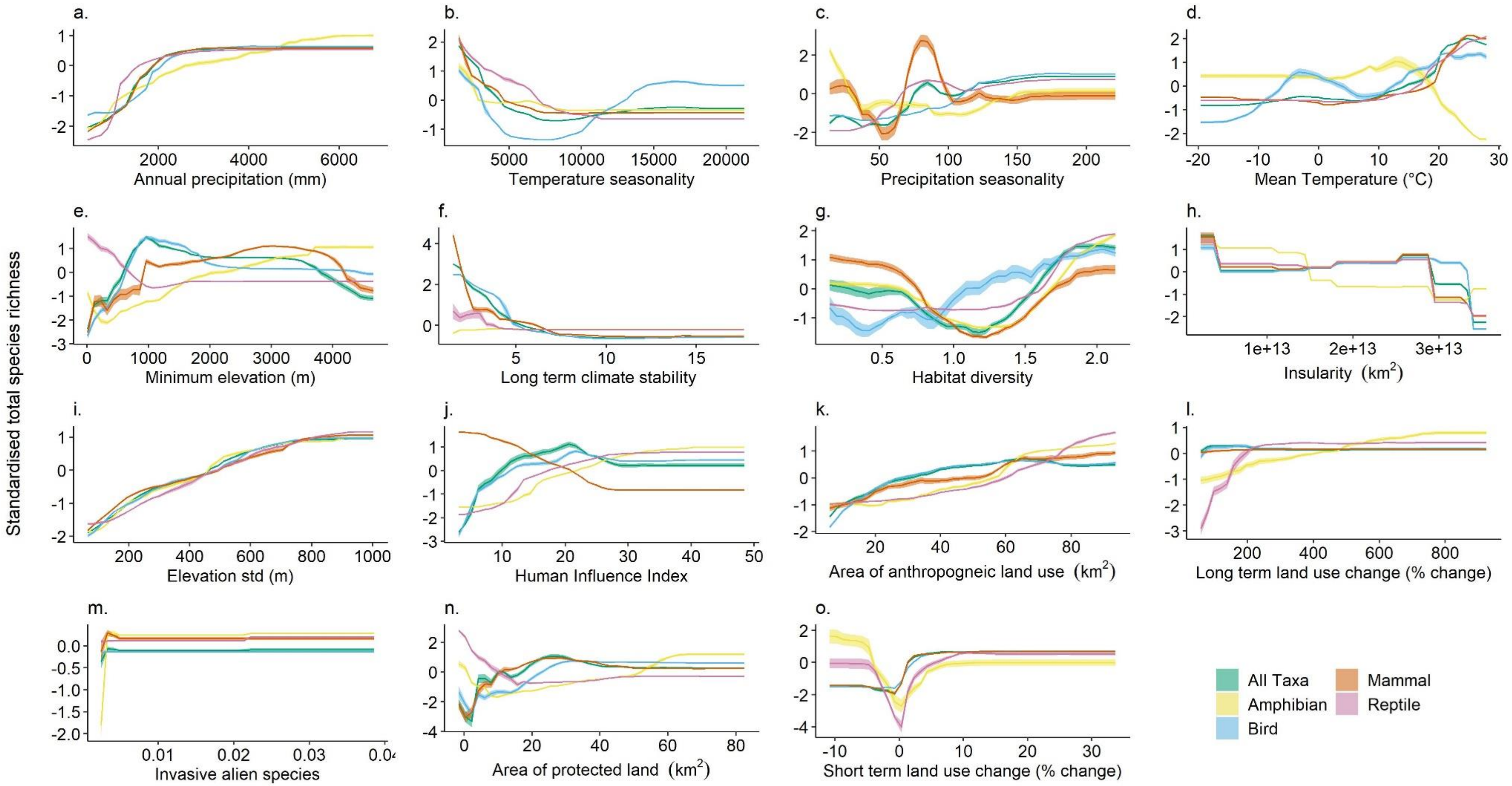

Supplementary Figure 10: Partial residual plots from global models of threatened amphibian, reptile, bird, and mammal species richness. Lines indicate the mean partial relationship between variables and threatened species richness from across 10 random forest models. The x-axis is limited to the central 90% of a variable's range. Shaded areas indicate the standard deviation around the mean partial relationship. Colours of both lines and shaded areas indicate taxonomic group: green = all taxa, yellow= amphibians, blue = birds, orange = mammals, and pink = reptiles. To aid comparison, responses have been scaled to have a mean of one and a standard deviation of zero. Source data are provided as a Source Data file.

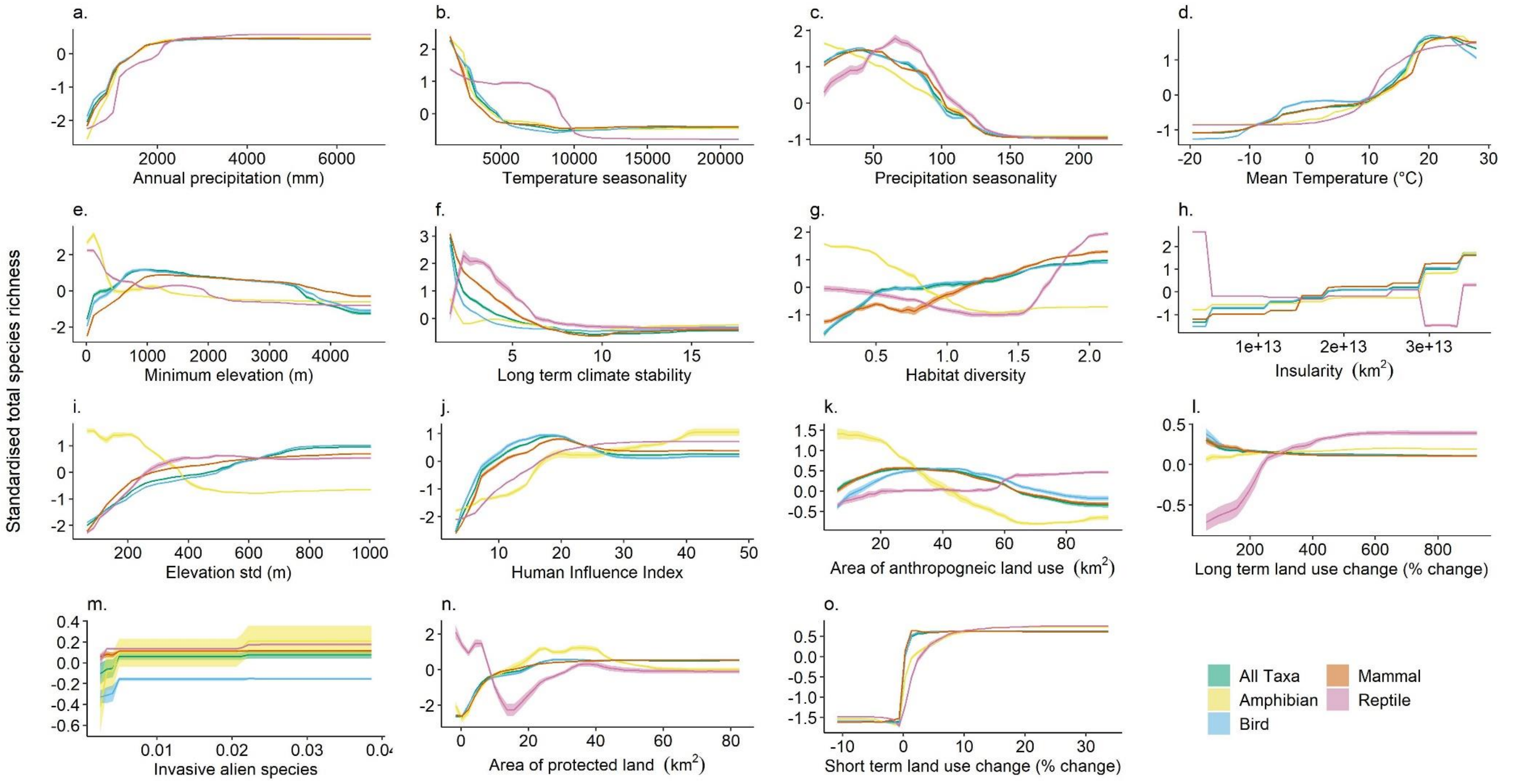

Supplementary Figure 11: Partial residual plots from global models of total amphibian, reptile, bird, and mammal species richness. Lines indicate the mean partial relationship between variables and threatened species richness from across 10 random forest models. The x-axis is limited to the central 90% of a variable's range. Shaded areas indicate the standard deviation around the mean partial relationship. Colours of both lines and shaded areas indicate taxonomic group: green = all taxa, yellow= amphibians, blue = birds, orange = mammals, and pink = reptiles. To aid comparison, responses have been scaled to have a mean of one and a standard deviation of zero. Source data are provided as a Source Data file.

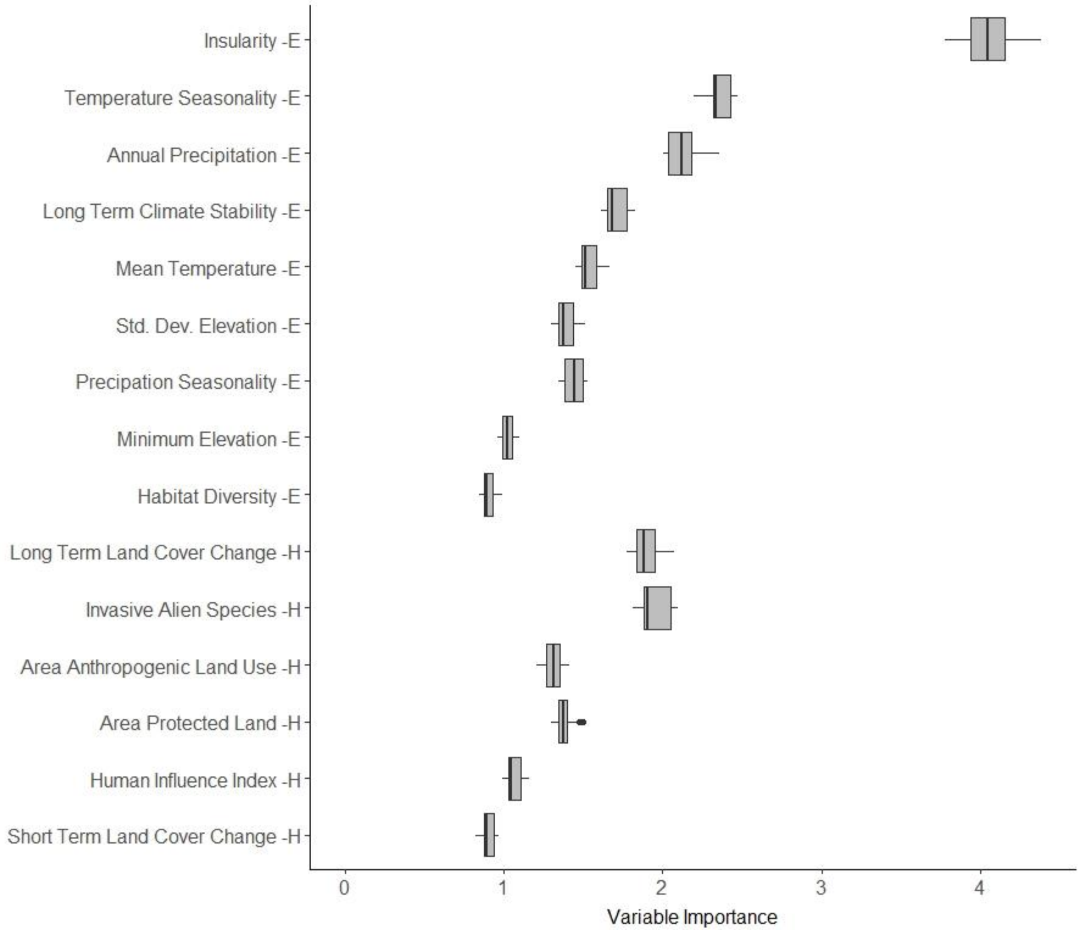

Supplementary Figure 12: Importance of individual variables in global models of residual threatened vertebrate species richness. The response in these models is the mean residual threatened species richness from models based on total species richness alone. Models performed well (mean R^2 = 0.83, S.D. ± 0.03). Variables are grouped into broader classes, which are indicated by the capital letters on the side of the variable names: Environmental (E) and Human Impact (H) covariates. Note, these models do not include total species richness as a covariate. Variables are ordered from top to bottom first by group, and then by their importance in the global model of threatened vertebrate species richness presented in the main results, where the modelled response is the number of threatened species in a grid cell. The line across each box indicates the median and the box boundaries indicate the interquartile range (IQR). Whiskers identify extreme data points that are not more than 1.5 times the IQR on both sides; the dots are more extreme outliers. Source data are provided as a Source Data file.

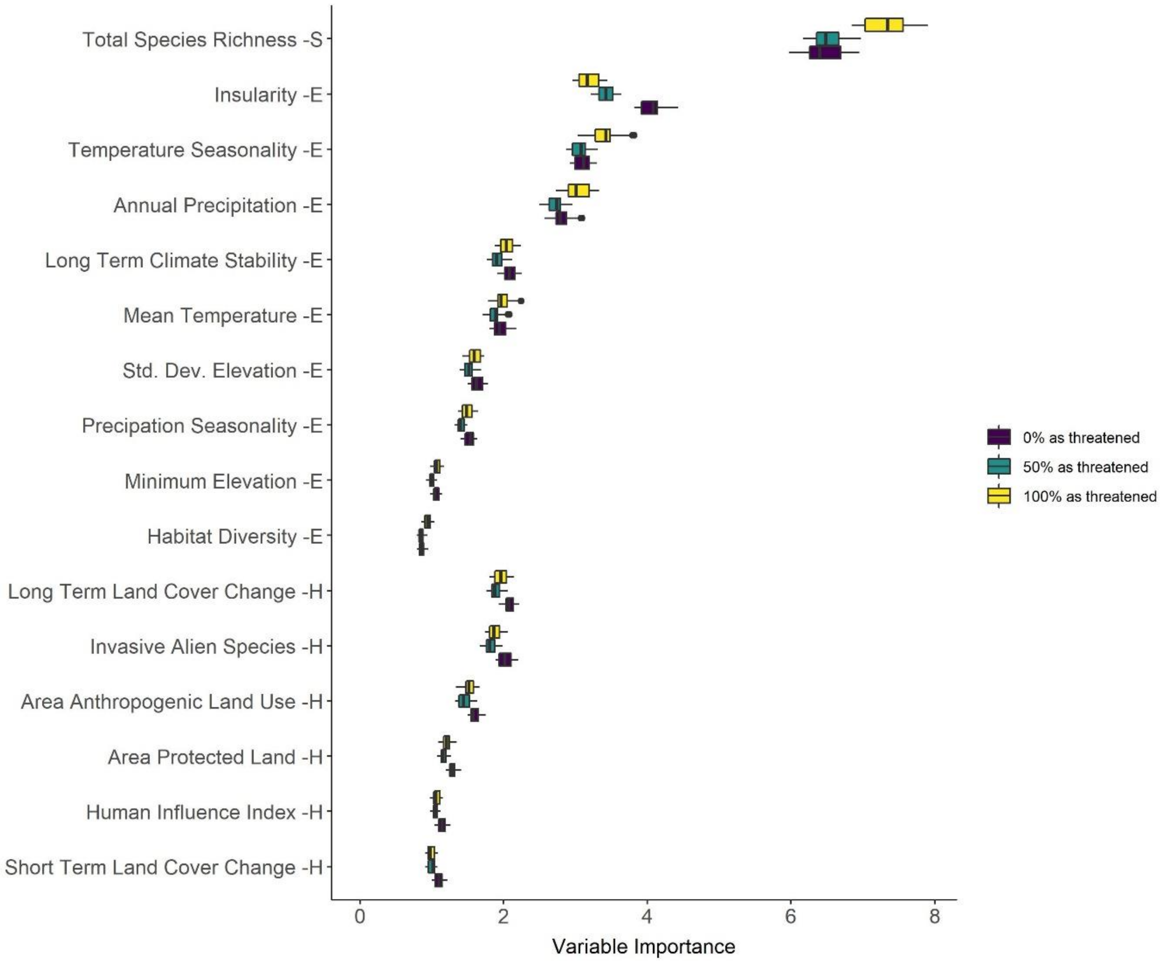

Supplementary Figure 13: Importance of individual variables for predicting global threatened vertebrate species richness with different assumptions of threat status for data deficient species. The response in these models incorporate DD species under three different assumptions of threat status (0%, 50% or 100% DD species classified as threatened). Shading of bars indicates the different assumption of threat status (0% = purple, 50% = green and 100% = yellow). Variables are grouped into three broad categories, indicated by the capital letters on the side of the variable names: Total Species Richness (S), Environmental (E), and Human Impact (H) covariates. Variables are ordered from top to bottom first by group, and then by their importance in the global model of threatened vertebrate species richness presented in the main results, where the response is the number of threatened species in a grid cell. The line across each box indicates the median and the box boundaries indicate the interquartile range (IQR). Whiskers identify extreme data points that are not more than 1.5 times the IQR on both sides; the dots are more extreme outliers. Source data are provided as a Source Data file.

Supplementary Table 1: Final parameters and performance of threatened species richness models fitted at global and regional scales using total species richness and a range of environmental and human impact covariates.

	Total vertebrates					Amphibians					Reptiles					Birds					Mammal				
**Region**	**n**	**R^2**	**±SD**	**nt**	**m**	**n**	**R^2**	**±SD**	**nt**	**m**	**n**	**R^2**	**±SD**	**nt**	**m**	**n**	**R^2**	**±SD**	**nt**	**m**	**n**	**R^2**	**±SD**	**nt**	**m**
**Global**	10	0.94	0.01	1000	3	10	0.72	0.05	1500	3	10	0.87	0.02	1000	3	10	0.92	0.01	1000	3	10	0.94	0.01	1000	3
**African**	10	0.85	0.03	1000	3	7	0.50	0.14	2000	3	10	0.78	0.07	1000	3	10	0.88	0.03	1000	3	10	0.77	0.03	1000	3
**Amazonian**	10	0.79	0.06	2000	3	10	0.67	0.11	1500	3	10	0.80	0.05	1500	3	10	0.84	0.05	1000	3	10	0.74	0.05	1500	3
**Arctico-Siberian**	10	0.95	0.01	1000	3	0	-	-	-	-	0	-	-	-	-	10	0.96	0.01	1000	3	10	0.80	0.04	1000	3
**Australian**	10	0.85	0.08	1500	3	10	0.78	0.14	1500	2	9	0.74	0.17	1500	3	10	0.77	0.09	1500	3	10	0.81	0.05	1500	3
**Chinese**	10	0.87	0.07	1500	3	10	0.79	0.12	1000	3	10	0.72	0.13	2000	3	8	0.81	0.05	1500	3	10	0.82	0.13	1500	3
**Eurasian**	10	0.89	0.02	1000	3	10	0.53	0.11	2000	3	10	0.63	0.11	1500	3	10	0.92	0.02	1000	3	10	0.79	0.04	1000	3
**Guinea-Congo**	10	0.84	0.07	1500	3	9	0.64	0.17	2500	3	10	0.83	0.08	1500	3	10	0.86	0.07	1500	3	10	0.81	0.08	1500	3
**Indo-Malayan**	10	0.87	0.04	1000	3	10	0.66	0.16	1500	3	10	0.63	0.17	1500	3	10	0.78	0.11	1500	3	10	0.94	0.03	1000	3
**Japanese**	8	0.43	0.13	2000	3	3	0.70	0.39	2000	3	0	-	-	-	-	7	0.68	0.18	2000	3	9	0.60	0.19	2000	3
**Madagascan**	10	0.76	0.16	1500	3	9	0.59	0.12	1500	3	10	0.71	0.12	1500	3	10	0.84	0.09	1500	3	9	0.78	0.14	2500	3
**Mexican**	10	0.83	0.06	1500	3	10	0.74	0.06	1500	3	8	0.64	0.15	2000	3	10	0.66	0.11	1500	3	10	0.74	0.16	1500	3
**North American**	10	0.66	0.04	1000	3	10	0.57	0.12	1500	3	10	0.60	0.19	2000	3	10	0.62	0.07	1500	3	10	0.77	0.07	1000	3
**Novozelandic**	9	0.77	0.10	1500	3	6	0.82	0.06	1500	3	6	0.62	0.25	2000	3	10	0.63	0.17	1500	3	10	0.85	0.11	1500	3
**Oriental**	10	0.86	0.06	1500	3	10	0.65	0.10	1500	3	10	0.84	0.05	1000	3	10	0.83	0.04	1500	3	10	0.90	0.03	1000	3
**Panamanian**	10	0.67	0.09	2000	3	10	0.64	0.14	1500	3	8	0.54	0.13	2000	3	10	0.64	0.13	1500	3	10	0.68	0.17	2000	3
**Papua-Melanesian**	9	0.81	0.09	1500	3	0	-	-	-	-	0	-	-	-	-	10	0.73	0.16	1500	3	9	0.74	0.11	2000	3
**Saharo-Arabian**	10	0.77	0.04	1000	3	8	0.42	0.09	2500	3	10	0.74	0.09	1000	3	10	0.70	0.04	1500	3	10	0.73	0.06	1500	3
**South American**	10	0.93	0.01	1000	3	10	0.63	0.05	1500	3	10	0.64	0.15	2000	3	10	0.90	0.02	1000	3	10	0.90	0.02	1000	3
**Tibetan**	10	0.94	0.02	1000	3	10	0.83	0.09	1500	3	10	0.82	0.09	1500	3	10	0.83	0.05	1500	3	10	0.92	0.04	1500	3

N indicates the number of random forest models fitted with an $R^2 \geq 0.25$, R^2 is the mean coefficient of determination across those models, S.D. is the standard deviation of those R^2 values, whilst nt is the number of trees and m the numbers of predictors used to build each regression tree that form the random forests. Source data are provided as a Source Data File.

Supplementary Table 2: Performance of models of threatened species richness fitted at the global scale with only total species richness as a predictor.

Taxa	Mean R^2	±SD	nt	m
Amphibian	0.04	0.03	1500	1
Bird	0.37	0.08	1000	1
Mammal	0.36	0.05	1000	1
Reptile	0.45	0.04	1000	1
Total Vertebrates	0.49	0.03	1000	1

Mean R^2 is the mean coefficient of determination across 10 random forest models, SD is the standard deviation of those R^2 values, whilst nt is the number of trees and m the numbers of predictors used to build each regression tree that form the random forests. Source data are provided as a Source Data File.

Supplementary References

1. Hijmans, R. J., Cameron, S. E., Parra, J. L., Jones, P. G. & Jarvis, A. Very high resolution interpolated climate surfaces for global land areas. *Int. J. Climatol.* **25**, 1965–1978 (2005).
2. Currie, D. J. Energy and Large-Scale Patterns of Animal- and Plant-Species Richness. *Am. Nat.* **137**, 27–49 (1991).
3. Luo, Z. *et al.* Environmental effects on vertebrate species richness: testing the energy, environmental stability and habitat heterogeneity hypotheses. *PLoS One* **7(4)**, e35514 (2012).
4. Davies, R. G. *et al.* Topography, energy and the global distribution of bird species richness. *Proc. R. Soc. B Biol. Sci.* **274**, 1189 LP – 1197 (2007).
5. Algar, A. C., Kharouba, H. M., Young, E. R. & Kerr, J. T. Predicting the future of species diversity: macroecological theory, climate change, and direct tests of alternative forecasting methods. *Ecography (Cop.).* **32**, 22–33 (2009).
6. Anderson, M. G. *et al.* Case studies of conservation plans that incorporate geodiversity. *Conserv. Biol.* **29**, 680–691 (2015).
7. Danielson, J. J. & Gesch, D. B. *Global multi-resolution terrain elevation data 2010 (GMTED2010)*. (2011).
8. Magurran, A. E. Measuring biological diversity. *African J. Aquat. Sci.* **29**, 285–286 (2004).
9. Kier, G. *et al.* A global assessment of endemism and species richness across island and mainland regions. *Proc. Natl. Acad. Sci.* **106**, 9322–9327 (2009).
10. Harrison, S., Viers, J. H., Thorne, J. H. & Grace, J. B. Favorable environments and the persistence of naturally rare species. *Conserv. Lett.* **1**, 65–74 (2008).
11. Harrison, S. & Noss, R. Endemism hotspots are linked to stable climatic refugia. *Ann. Bot.* **119**, 207–214 (2017).
12. Keppel, G. *et al.* Refugia: identifying and understanding safe havens for biodiversity under climate change. *Glob. Ecol. Biogeogr.* **21**, 393–404 (2012).
13. Singarayer, J. S. & Valdes, P. J. High-latitude climate sensitivity to ice-sheet forcing over the last 120 kyr. *Quat. Sci. Rev.* **29**, 43–55 (2010).

14. Davies-Barnard, T., Ridgwell, A., Singarayer, J. & Valdes, P. Quantifying the influence of the terrestrial biosphere on glacial–interglacial climate dynamics. *Clim. Past* **13**, 1381–1401 (2017).

15. Voskamp, A., Baker, D. J., Stephens, P. A., Valdes, P. J. & Willis, S. G. Global patterns in the divergence between phylogenetic diversity and species richness in terrestrial birds. *J. Biogeogr.* **44**, 709–721 (2017).

16. Cincotta, R. P., Wisnewski, J. & Engelman, R. Human population in the biodiversity hotspots. *Nature* **404**, 990–992 (2000).

17. Radeloff, V. C. *et al.* Housing growth in and near United States protected areas limits their conservation value. *Proc. Natl. Acad. Sci.* **107**, 940–945 (2010).

18. Stein, B. A., Scott, C. & Benton, N. Federal lands and endangered species: the role of military and other federal lands in sustaining biodiversity. *AIBS Bull.* **58**, 339–347 (2008).

19. Wilcove, D. S., Rothstein, D., Dubow, J., Phillips, A. & Losos, E. Quantifying Threats to Imperiled Species in the United States: Assessing the relative importance of habitat destruction, alien species, pollution, overexploitation, and disease. *Bioscience* **48**, 607–615 (1998).

20. Di Marco, M., Venter, O., Possingham, H. P. & Watson, J. E. M. Changes in human footprint drive changes in species extinction risk. *Nat. Commun.* **9**, 4621 (2018).

21. Ramankutty, N. & Foley, J. A. Estimating historical changes in global land cover: Croplands from 1700 to 1992. *Global Biogeochem. Cycles* **13**, 997–1027 (1999).

22. Millennium Ecosystem Assessment. *Ecosystems and human well-being: Biodiversity Synthesis*. (2005).

23. Turbelin, A. J., Malamud, B. D. & Francis, R. A. Mapping the global state of invasive alien species: patterns of invasion and policy responses. *Glob. Ecol. Biogeogr.* **26**, 78–92 (2017).
