# Supplementary material for: A global assessment of the drivers of threatened terrestrial species richness
Source: Nat Commun. 2020 Feb 20;11:993. doi: 10.1038/s41467-020-14771-6 (PMC7033199; doi:10.1038/s41467-020-14771-6)
Supplement: Supplementary file 3 — Reporting Summary [file 41467_2020_14771_MOESM3_ESM.pdf]

natureresearch

Corresponding author(s): NCOMMS-19-15718B

Last updated by author(s): Jan 14, 2020

Reporting Summary

Nature Research wishes to improve the reproducibility of the work that we publish. This form provides structure for consistency and transparency in reporting. For further information on Nature Research policies, seeAuthors & Referees and theEditorial Policy Checklist .

Statistics

For all statistical analyses, confirm that the following items are present in the figure legend, table legend, main text, or Methods section.

n/a	Confirmed	
☐	☒	The exact sample size (*n*) for each experimental group/condition, given as a discrete number and unit of measurement
☒	☐	A statement on whether measurements were taken from distinct samples or whether the same sample was measured repeatedly
☐	☒	The statistical test(s) used AND whether they are one- or two-sided<br>*Only common tests should be described solely by name; describe more complex techniques in the Methods section.*
☐	☒	A description of all covariates tested
☐	☒	A description of any assumptions or corrections, such as tests of normality and adjustment for multiple comparisons
☐	☒	A full description of the statistical parameters including central tendency (e.g. means) or other basic estimates (e.g. regression coefficient) AND variation (e.g. standard deviation) or associated estimates of uncertainty (e.g. confidence intervals)
☐	☒	For null hypothesis testing, the test statistic (e.g. *F*, *t*, *r*) with confidence intervals, effect sizes, degrees of freedom and *P* value noted<br>*Give P values as exact values whenever suitable.*
☒	☐	For Bayesian analysis, information on the choice of priors and Markov chain Monte Carlo settings
☒	☐	For hierarchical and complex designs, identification of the appropriate level for tests and full reporting of outcomes
☐	☒	Estimates of effect sizes (e.g. Cohen's *d*, Pearson's *r*), indicating how they were calculated

Our web collection on statistics for biologists contains articles on many of the points above.

Software and code

Policy information about availability of computer code

Data collection	No software was used. All datasets used in this study came from published sources.
Data analysis	All data analysis was performed in R Version 3.3.1.

For manuscripts utilizing custom algorithms or software that are central to the research but not yet described in published literature, software must be made available to editors/reviewers. We strongly encourage code deposition in a community repository (e.g. GitHub). See the Nature Research guidelines for submitting code & software for further information.

Data

Policy information about availability of data

All manuscripts must include a data availability statement. This statement should provide the following information, where applicable:

- Accession codes, unique identifiers, or web links for publicly available datasets
- A list of figures that have associated raw data
- A description of any restrictions on data availability

The species richness data generated and analysed during this study are included in the Source Data files. The other datasets utilised in this study are derived from published sources, cited in the methods section. The source data underlying Figs 1–4, Supplementary Figs 1-13, Tables 1-2, and Supplementary Tables 1-2 are provided as a Source Data file.

October 2018

Field-specific reporting

Please select the one below that is the best fit for your research. If you are not sure, read the appropriate sections before making your selection.

☐ Life sciences ☐ Behavioural & social sciences ☒ Ecological, evolutionary & environmental sciences

For a reference copy of the document with all sections, see nature.com/documents/nr-reporting-summary-flat.pdf

Ecological, evolutionary & environmental sciences study design

All studies must disclose on these points even when the disclosure is negative.

Study description	Here we assess the role of a suite of environmental factors and anthropogenic covariates for driving global patterns of threatened terrestrial vertebrate species richness. Using data on the distribution of amphibians, reptiles, birds and mammals, available from the IUCN and BirdLife International, we calculate global threatened species richness patterns both for individual taxonomic groups and all terrestrial vertebrates combined. We collate data on global environmental conditions (including climate, habitat diversity, elevation, and insularity) and on anthropogenic threatening processes (invasive alien species, land cover change, human population and land use) from a range of published sources. We then use a machine learning technique to assess the relative importance of these variables in driving global patterns of threatened species richness.
Research sample	We used data on the global distribution of all vertebrate species currently available from the IUCN or BirdLife International. This includes data on 26,746 species of amphibians, reptiles, birds and mammals, 5,341 of which are classified as 'threatened with extinction'(Red List status of critically endangered, endangered, or vulnerable) by the IUCN on the 2016 version of the Red List . We acquired global distribution data for amphibians,reptiles, and mammals from the IUCN, whilst distribution data for birds were acquired from BirdLife International. Data were available as spatial polygons of distribution boundaries, which we projected onto a Behrman equal-area projection and converted onto a grid with a cell size of 0.5° at 30°N and 30°S latitude.
Sampling strategy	For this study we used all range data for the global distributions of terrestrial vertebrates available from either the IUCN or BirdLife International. We classify threatened species as those species with an IUCN Red List status of critically endangered, endangered, or vulnerable. This provided an overall sample size of 5,341 threatened species.
Data collection	All of the datasets used in this study are from published sources, cited in the methods.
Timing and spatial scale	Data on species' distributions and threat status were acquired from the IUCN and BirdLife International in May 2017. These data use all available data and are constantly being updated. Covariate data were chosen based on the most relevant time period for our analysis. For example, data on climatic variables were acquired for the time period 1960-1990, a period that broadly coincides with the majority of data collection for the species' distribution data. All data were acquired an analyzed at a global scale.
Data exclusions	Analyses were initially performed at a global scale and then later repeated at the level of individual biogeogrphic realms (as defined by Holt et al., 2013, Science). At this second stage we excluded the Polynesian realm, as this realm consists of less than 10 data points (grid cells of approximately 50km2), and provides an insufficient sample size to fit useful models of species richness.
Reproducibility	For all levels of the analysis (either global, total vertebrate, or split by realm or taxonomic group), to ensure that our results were robust we fitted 10 models of threatened species richness and tested their performance on a withheld semi-independent subset of the data. To ensure wider reproducibility of our analysis, all code, including package versions, are available on GitHub (https://github.com/christinehoward399/Global-Rarity).
Randomization	Initially, the analysis was performed using all available data for the distribution of threatened terrestrial vertebrates. Species were organized by taxonomy and split into four major taxonomic groups (amphibians, reptiles, birds and mammals). Analyses were first performed at a global scale, but then split into 20 biogeographic realms as defined by Holt, B. G. et al. (Science, 2013).
Blinding	Blinding was not relevant for our study as we only used data from published sources and made no specific a priori hypotheses.

Did the study involve field work? ☐ Yes ☒ No

Reporting for specific materials, systems and methods

We require information from authors about some types of materials, experimental systems and methods used in many studies. Here, indicate whether each material, system or method listed is relevant to your study. If you are not sure if a list item applies to your research, read the appropriate section before selecting a response.

Materials & experimental systems

n/a	Involved in the study	
☒	☐	Antibodies
☒	☐	Eukaryotic cell lines
☒	☐	Palaeontology
☒	☐	Animals and other organisms
☒	☐	Human research participants
☒	☐	Clinical data

Methods

n/a	Involved in the study	
☒	☐	ChIP-seq
☒	☐	Flow cytometry
☒	☐	MRI-based neuroimaging
